# Heating Induced Nanoparticle Migration and Enhanced Delivery in Tumor Treatment Using Nanotechnology

**DOI:** 10.3390/bioengineering11090900

**Published:** 2024-09-07

**Authors:** Qimei Gu, Liang Zhu

**Affiliations:** Mechanical Engineering Department, University of Maryland Baltimore County, Baltimore, MD 21250, USA; qg1@umbc.edu

**Keywords:** local heating, whole-body heating, nanoparticle distribution, magnetic nanoparticle hyperthermia, nanoparticle migration, tumor, drug delivery, enhanced delivery

## Abstract

Nanoparticles have been developed as imaging contrast agents, heat absorbers to confine energy into targeted tumors, and drug carriers in advanced cancer treatment. It is crucial to achieve a minimal concentration of drug-carrying nanostructures or to induce an optimized nanoparticle distribution in tumors. This review is focused on understanding how local or whole-body heating alters transport properties in tumors, therefore leading to enhanced nanoparticle delivery or optimized nanoparticle distributions in tumors. First, an overview of cancer treatment and the development of nanotechnology in cancer therapy is introduced. Second, the importance of particle distribution in one of the hyperthermia approaches using nanoparticles in damaging tumors is discussed. How intensive heating during nanoparticle hyperthermia alters interstitial space structure to induce nanoparticle migration in tumors is evaluated. The next section reviews major obstacles in the systemic delivery of therapeutic agents to targeted tumors due to unique features of tumor microenvironments. Experimental observations on how mild local or whole-body heating boosts systemic nanoparticle delivery to tumors are presented, and possible physiological mechanisms are explored. The end of this review provides the current challenges facing clinicians and researchers in designing effective and safe heating strategies to maximize the delivery of therapeutic agents to tumors.

## 1. Introduction

It is estimated by the American Cancer Society that more than 2 million new cancer cases will be diagnosed and 611,720 cancer related deaths occur in 2024 [1]. Traditional treatment methods such as surgery, radiation, and chemotherapy have increased the survival rate in cancer patients via early diagnosis and intervention with advanced technology. Unfortunately, it is still a challenge to damage all tumor tissue at its original site. The remaining surviving tumor cells may metastasize, contributing to the majority of cancer deaths. 

Traditional cancer treatments often involve intravenous injection of therapeutic agents into the bloodstream. Once a drug is in the interstitial fluid space, diffusion to the tumor cells away from capillaries is difficult. Although there are different-sized therapeutic agents, their diffusion coefficients in fluid are usually not high. Recent studies showed that the diffusion coefficients of various small-molecule drugs are at the order of magnitude of 10^−10^ m^2^/s [2]. For large-sized therapeutic agents, their diffusion coefficients are even smaller. One example is the diffusion coefficient of nanoparticles in unbound interstitial fluid, which is only 6.7 × 10^−11^ m^2^/s [3]. The typical porosity of 0.2 in most tumors further limits the diffusion coefficient in the interstitial fluid space to approximately 14% of that in unbound fluid [3]. Other hurdles include the deposition of therapeutic agents on the surfaces of tumor cells and difficulty entering tumor cells. Thus, although potent therapeutics have been developed, delivering them to tumors and achieving sufficient and optimized drug concentrations in the entire tumor are significant challenges facing clinicians and bioengineers.

In the past decades, newly developed nanotechnology has advanced cancer treatment for better patient outcomes. Nanoparticles were studied as heat generators to confine energy in targeted tumors [4,5,6,7,8,9,10,11], as imaging contrast agents to visualize drug distribution [12,13,14,15,16,17], and as drug carriers to maximize drug delivery [17,18,19,20,21]. Among these functions, nanoparticles act as energy absorbers/generators to confine heating in targeted tumor regions while minimizing collateral thermal damage to surrounding tissue. Laser photothermal therapy, high-intensity focused ultrasound, and magnetic nanoparticle hyperthermia all utilize nanoparticles to enhance energy generation in tumors to cause cell damage [17,22,23,24,25,26,27,28,29]. In drug delivery, therapeutic agents could be attached to the surfaces of nanostructures or placed inside the hollow space of nanostructures to reduce systemic toxicity. Once the drug reaches the tumor region, drug release can be implemented and controlled by external stimulators [30] to achieve a desirable drug concentration for a prolonged time duration, therefore avoiding an initial concentration burst. In recent years, nanoparticles have been developed to combine imaging-assisted diagnosis with thermal damage capability [17], or to use heating to release drugs encapsulated inside hollow particles [30,31,32]. Advanced nanoparticles also allow therapeutic agents, targeting moieties, contrast agents, etc., to be attached to their surfaces to serve multiple functions in cancer treatment [33,34].

Nanoparticle distribution and their concentration in tumors are critical to cancer treatment. When heating intensity is elevated to a level to cause irreversible damage to tumor cells in hyperthermia treatment using nanoparticles, a replicable and controllable nanoparticle distribution is often difficult to achieve. The initial nanoparticle distribution within a tumor may change due to particle migration; this would further alter the thermal dosage needed to damage the tumor. In systemic delivery, passive advection and the diffusion of nanoparticles are negatively impacted by the interstitial fluid pressure (IFP) in tumors [35,36]. Any approaches including mild heating to change the transport properties of the interstitial space to allow fluid and particles to easily pass through would enhance the delivery of therapeutic agents to tumors. 

The present work focuses on understanding how local or whole-body heating alters the transport properties in tumors, therefore leading to enhanced nanoparticle delivery or optimized nanoparticle distributions in tumors. First, an overview of cancer treatment and the development of nanotechnology in cancer therapy is introduced. Second, the importance of particle distribution in one of the hyperthermia approaches using nanoparticles in damaging tumors is discussed. How intensive heating during nanoparticle hyperthermia alters interstitial space structure to induce nanoparticle migration in tumors is evaluated. The next section reviews major obstacles in the systemic delivery of therapeutic agents to targeted tumors due to unique features of tumor microenvironments. Experimental observations on how mild local or whole-body heating boosts systemic nanoparticle delivery to tumors is presented, and possible physiological mechanisms are explored. At the end of this review, the current challenges facing healthcare professionals in designing effective and safe heating strategies to maximize the delivery of therapeutic agents to tumors are discussed.

## 2. Development of Nanotechnology in Medicine

Developments in nanotechnology in the past decades have resulted in broader impacts on society. When particle sizes are at the scale of nanometers, many physical mechanisms normally not observed at large scales become evident. This is the size scale when quantum effects cause unique properties of particles. The physical properties of materials at the nanoscale may differ significantly from those at larger size scales. 

The size of nanoparticles could affect their interaction with external energy sources such as lasers, ultrasound waves, or magnetic fields. Magnetic particles subject to an alternative magnetic field can also release energy in terms of heat. However, the intensity of the heat generation rate is influenced by different physical mechanisms when the particle size varies. When magnetic nanoparticles are at the micrometer scale, the mechanisms are primarily hysteresis or Eddy’s heating. Once the particles are at the nanoscale (<100 nm), they experience superparamagnetism due to Nèel’s relaxation and/or Brownian relaxation [19,37]. In the presence of an oscillating electromagnetic field of laser light, when the natural frequency of the surface plasmon resonance matches the frequency of the incident laser light, the conduction band of electrons in the metallic (gold or silver) nanoparticle undergoes a collective coherent resonant oscillation [38]. The excited electrons cool off rapidly by energy exchange with the nanoparticles inducing heat dissipation to be used in hyperthermia applications [17,39,40]. This has been widely used in laser photothermal therapy for confining laser energy absorption in tumors to maximize thermal damage. Another heating method using nanoparticles is in ultrasound. Recent studies have demonstrated that temperature elevations were more than doubled in high-intensity focused ultrasound in targeted tumors when magnetic nanoparticles or gold nanoparticles were used to enhance ultrasound energy absorption [27,28,29]. 

Before nanotechnology, typical therapeutic drugs injected into the bloodstream may have been captured quickly by the liver, spleen, and kidney. Thus, this results in very low drug concentrations in the bloodstream to tumors. Developed therapeutic drugs can be encapsulated inside hollow nanoparticles and these drugs can be released later once they are inside targeted tumors. Protein–drug conjugated nanoparticles are typically around 50 nm in diameter, resulting in a long half-life in vivo and consequently facilitating their delivery to the targeted tumor site [33,41,42]. Antibody-based targeting ligands have unique in vivo properties and high target specificities to certain receptors only overexpressed in tumors [33,43]. If more drugs can be delivered directly in the targeted tumors via nanocarriers, systemic toxicity to the healthy tissues or organs in the body would be minimized. 

Nanoparticles can change the profile of drug release by their unique characteristics. The tumor microenvironment has amplified enzymatic activity, acidic pH, reductive or oxidative states, and increased reactive oxygen species. In recent years, polymeric nanoparticles have been developed to take advantage of the changed homeostatic chemical equilibrium in tumors [44]. These biodegradable polymeric nanoparticles can be triggered by biochemical stimulations to allow controlled drug release. External physical triggers, such as the introduction of thermal, electrical, ultrasound, or magnetic energy to tumors, may cause a rupture of various components in nanocarriers. Previous experiments demonstrated drug release from liposomes when the tumor temperature was elevated slightly to 39–40 °C using localized magnetic or ultrasound heating.

Different kinds of nanoparticles are used in medicine. Liposome-based nanoparticles are spherical sacs formed by lipid bilayers with diameters varying from 50 to 500 nm [45]. Techniques such as extrusion, sonication, solvent injection, and reverse-phase evaporation are often used in industry to form liposome-based nanoparticles. One feature of liposome-based nanoparticles is the encapsulation of hydrophilic drug molecules. Hydrophobic and amphiphilic [46,47,48,49] drugs can be encapsulated into lipid solution before the formation of the shells of nanoparticles. Temperature-sensitive lipid bilayer spherical shells can rupture at a specific temperature to release the encapsulated drugs [31,32,50]. 

Polymeric nanoparticles consist of synthetic polymers to achieve a specific molecular weight, biodegradability, and hydrophilicity. The synthesis of polymeric nanoparticles uses nanoprecipitation, electrospray, and emulsification. The unique degradation curves of polymeric nanoparticles allow manipulation of drug release [51]. For hydrophobic drugs that would be quickly cleared by the liver or kidney, they can be encapsulated inside hydrophilic polymers to allow their delivery to targeted tumors [52]. Dendrimeric nanoparticles are another kind of polymer nanoparticle. They are a non-viral vector composed of polymers with a nanoparticle core of diameter less than 50 nm. Typically, citrate-stabilized gold nanoparticles are used as the core, followed by a ligand exchange reaction to replace the citrate molecules with the polymer dendrimer [33,34,53,54]. Dendrimeric nanoparticles have a unique scaffold structure that allows various combinatorial therapeutic attachments to serve multifunctional purposes. 

Further, interactions between nanoscale proteins and their receptors may be studied by visualization of their distributions and movements in cells. Nanoparticles, when manufactured as fluorescence carriers, may improve the imaging ability of fluorescence in biology and medicine [17]. In contrast to traditional organic-based fluorescent dyes and fluorescent proteins, fluorescence-labeled nanoparticles offer improved sensitivity and photostability. Nanoparticles themselves can also be imaged due to their nanomolar or micromolar sensitivity range that can be detected via imaging systems. Thus, nanoparticles with fluorescence imaging capability may contribute significantly to imaging at the cellular level in cancer treatment.

## 3. Nanoparticle Distribution in Tumors in Magnetic Nanoparticle Hyperthermia

The primary goal of hyperthermia in cancer treatment is to maximize the power deposition in targeted tumors. Hyperthermia treatment is typically defined as elevating tumor temperatures above 43 °C for up to several hours to achieve direct tumor cell apoptosis or necrosis [55]. In traditional hyperthermia methods such as microwave, laser, and ultrasound, waves have to penetrate through the superficial layer before they can reach the targeted tumor. During these processes, a large amount of energy is absorbed by the superficial layer, leading to collateral thermal damage to the healthy tissue [56]. Recently, three hyperthermia methods have utilized nanoparticles to confine thermal energy to tumors. Laser photothermal therapy uses gold nanorods/nanoshells to enhance laser energy absorption. HIFU (high-intensity focused ultrasound) studies have demonstrated enhanced ultrasound energy absorption in tumors with the presence of gold nanoparticles in tumors [27,28,29]. In magnetic nanoparticle hyperthermia, iron-based nanoparticles serve as heat generators when subject to an alternating magnetic field [10,11]. In these studies, nanoparticle distribution within the targeted tumor often determines the efficacy of the thermal therapy in cell damage. In this section, we focus our review on one of the cancer treatments using magnetic nanoparticles to damage tumor tissue, with an emphasis on how local intensive heating changes tumoral transport properties, leading to dynamic particle migration. The experimental observations and theoretical simulations in magnetic nanoparticle hyperthermia could be applied to other hyperthermia treatments using nanoparticles. The general approach may also be used in drug delivery or imaging-assisted diagnosis to manipulate particle migration to desirable locations.

### 3.1. Heating Mechanisms of Magnetic Nanoparticle Hyperthermia

In magnetic nanoparticle hyperthermia, several physical mechanisms co-exist, and individual dominations depend on the size of the nanoparticles. The first mechanism is hysteresis loss in bulk and multi-domain magnetic materials [57]. However, when the particle size is less than 100 nm, superparamagnetic relaxation is dominant, specifically Nèel’s relaxation and Brownian relaxation [24,58]. Eddy’s current is another possible mechanism; however, its contribution is minor compared to other heating mechanisms [24]. 

### 3.2. Quantification of Heat Generation Rate Induced by Magnetic Nanoparticles

Previous theoretical study of the specific loss power (SLP) of a single nanoparticle has been derived to provide an expression to illustrate how the magnetic field strength, frequency, and the effective relaxation time determine the SLP induced by a single nanoparticle [24]. Recent experimental studies demonstrated that the expression shows the general trends; however, it may not be accurate when nanoparticles interact with each other, and with the surrounding tissue [24,59,60,61]. Thus, experimental measurements are the preferred approach to quantify the SLP induced by nanoparticles. 

The specific absorption rate (SAR), defined as the energy generation rate per unit mass of the tissue, is related to the SLP, defined as the energy generation rate per unit mass of iron (unit: W/kg Fe). The SAR value (unit: W/kg tissue) depends not only on the SLP value, but also on the nanoparticle concentration in the tissue. Volumetric heat generation rate *q*‴ typically used in the Pennes bioheat equation [62] is defined as the energy generation rate per unit volume of the tissue and is directly related to the SAR value. The following equation shows the relationship between SAR and SLP [63]:

SAR = *C* × SLP (1)

where *C* is the ratio of the iron mass to the total sample mass and thus can be considered as the iron mass concentration in gel samples or tissue in magnetic nanoparticle hyperthermia studies.

Experimental measurements of the SLP varied significantly due to the geometries of nanoparticles and the strength and frequency of the exposed magnetic field [37,59,63,64,65,66,67,68,69,70,71,72,73]. Etheridge et al. [64] showed that the SLP is approximately 17 W/g of iron when the magnetic nanoparticles were subjected to an alternating magnetic field of 3 kA/m at a frequency of 215 kHz. When the magnetic field strength was at 40 kA/m at a frequency of 175 kHz, the SLP was determined to be much larger at 47–54 W/g [69]. LeBrun et al. [59] also quantified the SLP of a commercially available ferrofluid. It was found that their SLPs varied from 13 to 16 W/g when the nanoparticles were subjected to an alternating magnetic field of 5 kA/m at 190 kHz [59]. An impressive SLP of 104 W/g Fe was measured using a very strong magnetic field of 20 kA/m at a frequency of 829 kHz, again confirming the dependence of the SLP on magnetic field strength [70]. A recent study showed that adding a surface coating based on sodium citrate would double the SLP of Fe_3_O_4_ nanoparticles [71]. The latest SLPs are reported way above 200 W/g Fe when exposed to a magnetic field of 40 kA/m; however, they have a large frequency of 13.56 MHz [72]. Interestingly, a recent experiment implemented a magnetic field with a frequency in the GHz range, leading to an ultra-fast temperature rise rate over 100 K/s [73]. The large variations in these measured SLPs demonstrate the importance of experimental approaches of determining the SLP. Once the nanoparticles are manufactured, the local nanoparticle concentration distribution *C* is an important parameter to determine needed dosage to cause irreversible thermal damage to the entire tumor.

### 3.3. Experimental Studies in Magnetic Nanoparticle Migration during Hyperthermia

Animal experiments are commonly used before clinical trials to test the treatment efficacy of magnetic nanoparticle hyperthermia in cancer treatment. These experimental studies have demonstrated the capability of elevating tumor temperature above 50 °C using a relatively low strength of magnetic field. In one study, a tumor grown from a Tu212 cell line on a mouse was heated easily to 50 °C after injecting 0.5 mL of a 5.8% ferrofluid into the tumor [74]. Similar results were found in xenograft liver tumors (SMMC-7721 cell line) implanted in mice [75]. Attaluri et al. performed heating experiments [76] on PC3 tumors implanted on mice, using an alternating magnetic field of 5 kA/m, and recorded temperature elevations above 60 °C [76]. In LeBrun et al. [10,11], they designed a heating protocol of heating for 25 min to PC3 tumors implanted in mice. Their results showed a complete disappearance of the damaged tumors three days after the heating treatment. Another research group used multiple sessions of magnetic nanoparticle hyperthermia on prostate cancer implanted in rats [77]. They observed tumor inhibition with the tumor growth less than 50% of that in the untreated tumors [77]. Similar tumor growth inhibition was reported by another group after magnetic nanoparticle hyperthermia treatment of implanted MiaPaCa02 human xenograft tumors [78], as well as in pancreatic ductal adenocarcinoma [70]. In Li et al. [79], they reported severe tissue damage in a human breast tumor (MCF-7 line) implanted in mice after magnetic nanoparticle hyperthermia. The destroyed tumor cells appeared structurally abnormal with fragmented nuclei. They also found that the higher the frequency of the exposed alternating magnetic field, the more significant the cell damage. A recent study [72] developed magnetic graphene oxide nanoheaters injected into glioma-bearing rats for heating treatment. The authors reported significant inhibition of tumor growth and tripled survival days in the heating group. These experimental data typically provided the total amount of nanoparticles in tumors; however, they rarely reported the nanoparticle distribution in the tumors as well as possible particle migration during the heating. 

In animal studies, the thermal dosage needed to completely damage tumors might be affected by the original nanoparticle concentration distribution in the tumors and possible particle redistribution. It is well known that high-intensity heating usually damages the blood vessels and cells in tissue. In direct intratumoral injections of nanofluid into tumors, nanoparticles are driven by the high pressure at the tip of the injecting needle. Once the injection stops, the nanoparticle distribution is found to have no noticeable change several hours after the injection, based on previous studies using transparent tissue equivalent agarose gels. Although local nanoparticle concentration near the injection site is usually high, the driving force due to diffusion has to overcome other barriers to move the nanoparticles to low-concentration regions. When tumor cells are completely damaged, the interstitial fluid space may be enlarged due to rupture of cells which releases inside fluid. This would increase tumor porosity and allow easier particle diffusion. During heating, nanoparticle migrations after tissue damage would change the volumetric heat generation rate that is related to nanoparticle distribution. Designing an effective heating protocol would require inclusion of dynamic migration in the theoretical simulation. 

**Figure 1:**
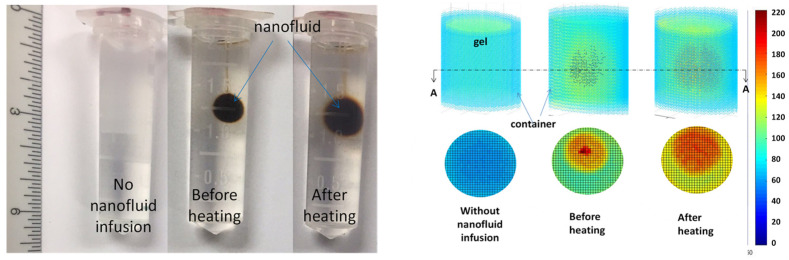
Nanoparticle migration in agarose gels: (**left** panel) photo of the specimen with or without ferrofluid injection; (**right** panel) microCT images of the gel specimen. The right vertical bar represents the microCT gray scale values. This figure is adopted from the PhD dissertation of the 1st author [81].

Experimental evidence of nanoparticle migration in tissue-equivalent agarose gel or animal tissue has been reported in recent years. One experiment was designed to compare iron-based magnetic nanoparticle distribution volumes inside agarose gels with or without local heating [80]. Shown in Figure 1, without a ferro-fluid injection of 0.2 mL, the agarose gel specimen is semi-transparent, while the gel specimen has a dark-colored spherical region represented by the presence of injected magnetic nanoparticles. The needle track is barely observed on the top of the middle panel. The third photo from left shows the same gel specimen after undergoing heating for 15 min, induced by an alternating magnetic field of 5 kA/m and frequency of 200 kHz. One can see that the nanoparticles occupy a bigger volume after the heating. However, since the nanoparticles may not be uniformly deposited in the gel after infusion, the darker-color region alone is not suitable to quantify nanoparticle spreading in gels.

A high-resolution microCT imaging system with pixel gray scale values from 0 to 255 was later used to image the density of the same agarose gel specimen with direct injections of nanoparticles [80,81]. The right panel in Figure 1 illustrates the 3D color contours of the microCT gray value distribution (top row), and gray value distribution on the cross-sectional plane A-A (bottom row). Three-dimensional visualization of the region with nanoparticle presence deviates from a spherical shape. The nanoparticle distribution inside the gel before heating is not uniform, varying from 220 at the center near the infusion site to 135 at the periphery. After heating, the region is gets bigger; however, the nanoparticles are less concentrated than before heating with a more uniform gray scale value of approximately 190. Based on ten gel specimens, the nanoparticle distribution volume is calculated as 0.58 ± 0.15 cm^3^ (mean ± SD, *n* = 10) using a cutoff gray scale value of 65, which is the average gray scale value of gels without nanoparticles. Based on 10 samples, the nanoparticle distribution volume increases after heating by 26% to 0.73 ± 0.14 cm^3^. Nanoparticle migration induced by heating was evident since the same gel specimen was scanned by microCT before and after local heating. 

Similarly, nanoparticle distribution as affected by high-intensity local heating could be studied using a microCT scan of tumor specimens [82]. An ideal situation is to scan the same tumor with a direct ferro-fluid injection before and after a local heating. This would require a costly microCT system accommodating a live animal bearing a tumor. In the past, some researchers used resected tumors with or without local heating and determined whether there was a statistical significance in the particle distribution volumes between these two tumor groups. 

**Figure 2:**
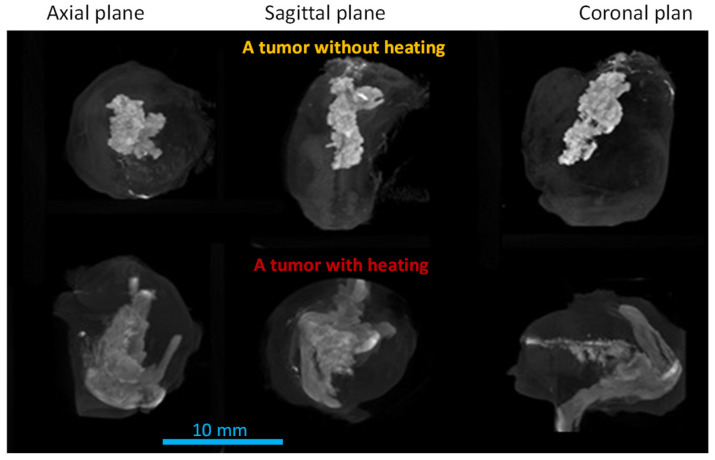
MIP images of two resected tumors in three projections. The tumors were injected with 0.1 cc ferrofluid. The top panel is from a tumor without heating and the bottom panel is a tumor resected and imaged after heating for 25 min. This figure is adapted from the PhD dissertation of the first author [81].

In one of the previous studies, two groups of resected PC3 tumors with or without heating were scanned by microCT [82]. Figure 2 gives the MIP (maximum intensity projection) images of PC3 tumors with an intratumoral nanoparticle injection. The white/gray clouds in the images represent the presence of nanoparticles in tumors. The three images on the top row represent a tumor with nanoparticles, but without heating treatment. They show very high-concentration regions at the tumor center, illustrated by the bright white regions. The bottom three images are from a tumor subjected to heating for 25 min. One notices that the nanoparticle deposition regions in the heated tumor are more irregular and occupy a larger volume than those in the tumor without heating. Since both tumors were injected with the same amount of iron-based nanoparticles, the larger volume occupied by the nanoparticles in the heated tumor suggested the possible spreading of nanoparticles from the central region to tumor periphery during heating. The nanoparticle concentration in the heated tumor is lower, implied by the less bright white color in the bottom images. 

Quantification of the distribution volume of nanoparticles at a specific Hounsfield range illustrates evidence of particle migration in PC3 tumors. The nanoparticle distribution volume in the highest particle concentration range (Hounsfield unit > 2000) in the heating group decreased by 22% compared to that of the control group [82]. On the contrary, the distribution volumes in the groups with lower particle concentration ranges all increased compared to the control, ranging from 16% to 91%. Overall, the total nanoparticle distribution volume in the tumor group heated for 25 min was 42% larger than that in the tumor group without heating, indicating possible particle migration induced by local high-intensity heating [82]. 

Nanoparticle migration to tumor center regions was also observed in pancreatic tumors (ductal adenocarcinoma) after hyperthermia treatment [70]. In this study, iron-based nanoparticles were injected intratumorally into a pancreatic ductal adenocarcinoma implanted in a heterotopic xenograft mouse model, and the tumor was subjected to an alternating magnetic field of 20 kA/m at 829 kHz. Through histological analyses of tumor sections post resection, the authors reported a greater presence of magnetic nanoparticles in the tumor central region than in the control without heating [70]. This suggested that nanoparticle migration to the tumor center is triggered by the heating, although the exact mechanisms behind the observation were unclear [70]. 

### 3.4. Theoretical Simulations to Understand Possible Mechanisms of Nanoparticle Migration during Heating

Theoretical simulation is a useful tool to design heating protocols for cancer patients before a heating treatment. In the past decades, the Pennes bioheat equation [62] has been used as the governing equation to model the thermal effects of local blood perfusion, metabolism, and external heat sources of hyperthermia approaches. Large disagreement between experimental results of temperature measurements and theoretical predictions was not uncommon [68,77] and this were attributed to inaccuracy of the nanoparticle concentration *C* or the energy absorption rate SAR, as well as a failure to include the dynamic responses of particle migration during heating.

Previous theoretical simulations of the tumor temperature field often assumed an unchanged nanoparticle distribution during heating. This may be directly contradictory to experimental observations. Previous experiments using transparent tissue equivalent agarose gels have reported almost no noticeable change in nanoparticle distribution several hours after the injection [80,83,84,85]. However, this equilibrium may be disrupted due to changes in the transport properties in tumors. A more uniform nanoparticle distribution after heating has been suggested by researchers due to more uniform temperature elevations being observed in their studied tumors when heating was repeated [86]. 

It is possible that local heating resulting in local thermal damage (the Arrhenius integral Ω) changed transport properties. Intensive local heating will elevate tissue temperatures to cause rupture of cell membranes and cell deaths. The originally bound intracellular fluid, once released, may increase porosity [3,87,88], as follows:

*ϕ*(*x*,*y*,*z*,*t*) = *ϕ_0_* + (80% − *ϕ_0_*)[1 - e^(−Ω(*x*,*y*,*z*,*t*)^)] (2)

where *ϕ_0_* is the original porosity of tumors before the heating, and Ω is the Arrhenius integral indicating the extent of thermal damage. This equation suggests larger porosities when the thermal damage is gets bigger. The 80% shown in Eq. 2 is the upper limit of porosity due to 20% of tumor volume being occupied by the extracellular matrix, which is assumed to not be affected by thermal damage [88]. 

Further, the diffusion coefficient *D_n_* is a function of the interstitial space fraction (porosity) *ϕ*:

*D_n_* = *D_n_*_,*f*_ [2*ϕ*/(3 - *ϕ*)] (3)

where *D_n_*_,*f*_ is the nanoparticle diffusion coefficient in interstitial fluid with the porosity equal to 1 [3,89,90,91]. This equation indicates how the diffusion coefficient could be enhanced by a large porosity in the tumor during heating. This equation suggests that when the interstitial space fraction increases from 20% in an unheated tumor to 80% after heating to completely damage the tumor cells, the diffusion coefficient could increase more than 5-fold.

Nanoparticle migration is usually modeled by nanoparticle diffusion in a porous medium [89,91]. The governing equation for the nanoparticle concentration (mol per unit volume of tissue) *C* is written as follows:

∂*C*/∂*t* = ∇•[*D_n_ ϕ* ∇(*C*/*ϕ*)] (4)

Finally, the dynamic nanoparticle concentration *C* would affect the volumetric heat generation rate in the SAR expression to influence the temperature elevations [88]. 

**Figure 3:**
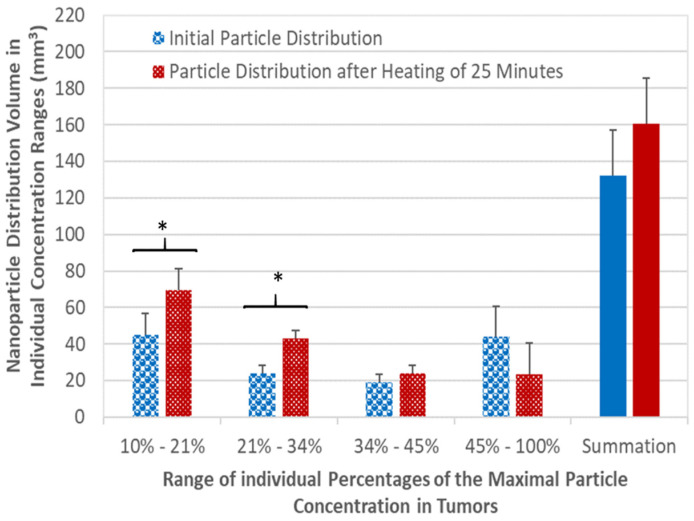
Nanoparticle distribution volumes in individual particle concentration ranges in tumors. The left bars of individual sets are the initial volumes and the right bars of individual sets are volumes after heating for 25 min. The solid bars on the far right side are the total nanoparticle distribution volume, the volume summation of all the individual ranges. The symbol * denotes a significant difference with the *p*-value less than 0.05 based on Student’s *t*-test and the Wilcoxon rank sum test. This figure is adapted from [88].

This approach has been tested in PC3 tumors in a magnetic nanoparticle hyperthermia study with a coupled simulation system [88]. Figure 3 illustrates nanoparticle distribution volumes in various concentration ranges. The bars on the left of individual sets are the initial nanoparticle distribution volumes. The bars on the right of individual sets denote the nanoparticle distribution volumes after heating for 25 min. The set on the right side of the figure shows how the total nanoparticle distribution volume changes from the initial 132 ± 35 mm^3^ to 160 ± 27 mm^3^ after 25 min of heating. In the highest particle concentration range (45–100% of the maximal concentration), the nanoparticle distribution volume decreases from the initial 44.2 ± 16.7 mm^3^ to 23.7 ± 17.6 mm^3^. On the contrary, one observes significant volume increases in the lower particle concentration ranges. The nanoparticle distribution volumes after heating increase by 28%, 78%, and 54% from their initial values, shown on the three sets of bars from the left. The theoretical predictions of the overall trend of nanoparticle migration in individual concentration ranges agrees very well with experimental analyses of microCT images of PC3 tumors [82].

Although the theoretical simulation results agree with the experimental data, enhancement in nanoparticle diffusion due to thermal damage may be one of the mechanisms behind nanoparticle migration. Several other factors including trans-tumoral fluid transport and lymphatic flow are not included in the model [88]. However, results from these studies may suggest the feasibility of manipulating nanoparticle dispersion to desirable tissue regions using controlled local heating.

## 4. Nanoparticle Delivery in Tumors Enhanced by Mild Heating

### 4.1. Nanoparticles as Drug Carriers 

The many nanostructures for drug delivery include liposomes, polymers, micelles, dendrimers, nanocrystals, nanorods, and drug–polymer conjugates [92,93]. Placing drug molecules inside nanostructures may minimize drug degradation and offer the possibilities of targeting and controlled release [94]. Nanocarrier–drug conjugates are effective and selective in comparison with the traditional formula of drugs. Nanocarriers may also reduce the toxicity level and other adverse effects in healthy tissues by depositing more drugs in the targeted sites. 

Most drugs are administered to the human body via intravenous injections [95]. After a systemic injection of drug-loading nanofluid into the vein, drug concentration in the bloodstream can be maintained at a high level depending on the drug’s circulating half-life. One of the biggest challenges is drugs accumulating in the liver or spleen [96]. The liver and spleen are part of the mononuclear phagocyte system. Their jobs are to filter toxins from the bloodstream. Therefore, the liver and spleen may prevent drugs from reaching their targeted tumor site [41]. 

Antibodies are often attached to drug-carrying nanoparticles to target specific tumors [97]. Active targeting antibodies and peptides can be anchored to the receptor structures often overexpressed at the targeted locations [98]. The main targets are usually receptors on cell membranes, antigens, or proteins on the shells of cells, and lipid components of the cell membranes. Typically, the tumors have specific receptors overexpressed. For example, it was reported that Ephrin type A receptor 2 is overexpressed at the surface of PC3 cells [99], while the receptor is at a low level in normal tissues. Thus, recombinant monoclonal antibody 1C1 can be used to bind the Ephrin type A receptors 2 on PC3 tumor cells [100]. In some studies, a strong magnetic field is used to guide magnetic nanoparticles to a desired tissue location when magnetic nanoparticles are used as nanocarriers [96]. The unique advantages of an external magnetic field are to remotely navigate nanoparticles to the targeted site. In passive targeting, the prepared drug carrier complex circulates around the entire body and is driven to the target site by attraction or binding influenced by properties such as temperature, pH, molecular site, and its shape. Unlike normal tissues, tumor capillaries are often leaky. Systemically administered nanoparticles have been shown to passively accumulate in tumors because of the enhanced permeability and retention effects [101].

### 4.2. Challenges in Drug Delivery

It is well known that tumor blood vasculature forms rapidly to result in abnormal branching patterns and lumen structures [102]. Blood perfusion supply to tumors can be lower than that to the surrounding normal tissue, especially in the late growing stages [103]. Decreased blood perfusion rate reduces oxygen supply to tumors and causes a hypoxic microenvironment. Hypoxia at the tumor central regions often boosts tumor metastases. Low blood perfusion also reduces the total amount of the systemically administered drugs to targeted tumors. 

Solid tumors show a higher-than-normal interstitial fluid pressure, which is another obstacle to transcapillary fluid flow. This barricade results in the ineffective uptake of therapeutic agents. Several factors may cause an increase in interstitial fluid pressure within the tumor site, such as vessel abnormalities, fibrosis, and shrinkage of the interstitial matrix [36]. Vascular abnormality may be due to the fast growth of tumors within a limited space in tissue [104]. The interstitial fluid space often occupies less than 15% of the body volume. Exchange of oxygen, nutrients, and waste occurs in the interstitial fluid space. Fluid exchange among capillaries, interstitial space, and lymphatic vessels is critical to the homeostasis of tissue [105,106]. A compromised lymphatic system in tumors cannot drain fluid out of tumors, and therefore results in IFP elevations within the tumor [107].

After extravasation, penetration of nanocarriers into the interstitial space is dominated by diffusion and advection of the carrier solution in the porous tissues. Nanoparticle parameters, such as size, shape, surface properties, and concentration within the bloodstream, are important factors that influence particle transport. The diffusion coefficient of nanoparticles can be as small as 9 × 10^−12^ m^2^/s within the extracellular space [3,108]. Based on this value, the drug/nanoparticles could only travel for less than 0.2 mm from the capillary surface after one hour. The diffusion and advection can be further compromised for the nanoparticles, since nanoparticles may stick on cell surfaces. Drug-carrying nanoparticles may not be able to continue to move with the fluid [109]. 

The mechanical properties of a tumor and its surrounding healthy tissue regulate how a growing tumor pushes the host tissue and, conversely, how the host tissue limits tumor growth [110]. Tissue-level stresses usually depend on the elastic modulus or stiffness of both the tumor and its hosting tissue [110]. They also may depend on myofibroblast-like fibroblasts compressing matrix components [111]. Collagen fibers actively resist tensile loads, which tend to restrict the expansion of nodular tumors. In general, the solid stress in a tumor is compressive in all directions [111]. Elevated solid stress in tumors also contributes to difficulty in nanoparticle diffusion and advection in the interstitial fluid space.

### 4.3. Mild Heating in Enhancing Systemic Drug Delivery in Tumors

As stated in Starling’s law, the major driving forces for transcapillary flow include the hydrostatic pressure difference and colloid osmotic pressure difference between the tumoral capillaries and the tumor interstitial space [89,112]. Previous experiments have reported low blood perfusion rates in tumors [113]. Unfortunately, the poor blood perfusion rates in tumors also lead to low hydrostatic pressure in tumoral capillaries. With the increased IFP in tumors, the driving force of transcapillary fluid transport is attenuated. Advection of nanofluid and particle diffusion in the interstitial fluid space are limited by the elevated internal pressure and poor blood perfusion in tumors. Any approaches to address these individual factors within the tumor’s microenvironment would reduce the barriers to promote drug-carrying nanofluid flow in tumors, to promote drug delivery. In systemic drug delivery, unfortunately, less than 4% of the therapeutic agents injected eventually reach targeted solid tumors [114]. To address this low payload, investigators have developed methods to prolong the circulation time of therapeutic agents in the bloodstream, and/or to attach a targeting moiety on nanocarrier surfaces for improved uptakes in tumor cells. Another method that was investigated in the past is to implement mild local or whole-body heating to increase drug delivery to tumors. Mild heating in cancer therapy is usually referred to non-lethal heating that would not cause irreversible thermal damage to tumor cells.

#### 4.3.1. Local or Whole-Body Heating on Blood Perfusion and IFPs in Tumors

Blood circulation plays an important role in dissipating heat during hyperthermia. Experimental studies by Song [115] showed a four-fold increase in the blood flow in the skin when subjected to heating at 43 °C for 60 min. On the contrary, their experiments reported a very limited initial blood perfusion increase and later decrease when the heating lasted for a long time [115]. Local heating may cause reversible thermal damage to blood vessels, therefore altering the local tumor microenvironment. Hauck et al. reported enhanced delivery of chimeric 125I-labeled 81C6 to gliomas implanted in mice with local heating for several hours [116]. In a study by Lammers et al. [117], local heating was introduced by a warm-water bath to tumor-bearing limbs, and a larger increase in copolymer delivery to one of the three tumor groups was reported [117]. A recent experimental study [118] demonstrated significant blood perfusion increases in PC3 tumors measured by a laser Doppler flowmeter. In this experiment, the implanted PC3 tumor was heated using an illuminator to elevate its temperature above 39 °C. The 1 h local heating to the PC3 tumors showed a modest increase of 20% in local blood perfusion rate from the baseline [118]. Using local mild hyperthermia, Fan et al. [119] reported tumor blood flow increases by local mild hyperthermia, and they also observed a 3-fold increase in nanoparticle delivery efficiency in tumors. In another study, strong local heating to 43.8 °C for a short duration of 4 min was given to 4T1 tumors, a murine mammary carcinoma, implanted on mice, immediately followed by a tail vein injection of nanofluid [120]. Retention of the nanoparticles (red fluorescence) in tumor tissues resected 24 h later increased by seven times compared with the tumors without heating. They attributed this retention to increases in the blood perfusion rate in the tumors, since they observed that more blood vessels in the tumors opened after the heating [120]. 

Previous experimental results on how local heating affected tumor IFP were not consistent. One study showed significant decreases in IFP after immersing the melanoma tumors in a water bath of 43 °C for 30–60 min [121]. Reduced IFPs in human breast tumors after the tumor temperature was elevated to 42 °C by laser heating were reported with enhanced deposition of liposomes in the tumors [122]. Another experiment by Gu et al. [118] focusing on local heating of PC3 tumors for one hour showed very limited IFP reductions from its pre-heating baseline values, approximately less than 2 mmHg immediately or two hours post heating. However, local heating of implanted gliomas at 42 °C for several hours showed no significant differences at any time point in the measured IFPs [123].

Mild whole-body hyperthermia has also been implemented to increase nanoparticle delivery to tumors. The first investigation was performed by Sen et al. [124]. In this study, the tumors used were murine colon tumors and murine melanomas implanted in mice. After the mice were subject to 2 h, 4 h, or 6 h of mild whole-body hyperthermia, they measured smaller IFPs in the tumors, when compared to the control group without heating. They also found that IFP reductions, increases in local tumoral blood perfusion, and reductions in tumor hypoxia were correlated. Winslow et al. [125] implemented a 4 h whole-body hyperthermia on human head and neck tumors implanted in mice. The IFPs in the tumors after 4 h of whole-body hyperthermia were 23% smaller than those in the tumors without heating, and the reductions lasted for more than 24 h. Fluorescent imaging of liposomes circulating in the bloodstream demonstrated more opened blood vessels and more intensive liposome extravasation in the frozen tumor sections. 

Gu et al. tested this approach to evaluate whether the results were the same on PC3 human prostate tumors implanted in mice [82,118]. They injected 0.2 mL of nanofluid containing gold nanoparticles via the tail vein of mice after 1 h or 4 h of whole-body hyperthermia. A micro-pressure transducer was inserted into the tumor tissue to measure IFPs repeatedly at the same tumor location. They reported significant decreases in IFPs in tumors in the heating groups from the values before the heating. On average, IFP reductions of 9.0 ± 6.3 mmHg and 4 ± 4.6 mmHg were measured in the tumor groups with whole-body hyperthermia for 1 h and 4 h, respectively [118]. The reductions in the IFPs were also sustained for 24 h in both groups. After the heating experiment, ICP-MS was used to assess the total amount of gold in the resected tumor, showing an increase in gold mass of 51–67% in the heating groups compared to that in the non-heating group. Since the IFP reduction in the 1 h heating was actually larger than that in the 4 h heating group and the enhancement in nanoparticle deliveries were similar, 1 h mild whole-body heating may be a readily implementable strategy in future clinical studies [118]. 

#### 4.3.2. Possible Mechanisms of Heating on Tumor Microenvironment

The specific mechanisms of mild whole-body heating with only several degrees Celsius of temperature elevations to facilitate drug/nanoparticle delivery to targeted tumors are still unclear. Previous studies have demonstrated that heat-induced permeability increases to boost the drug/nanostructure entering the interstitial space in tumors in systemic drug delivery [35,126,127,128,129]. One paper reviewed the effectiveness of combined radiofrequency thermal ablation and adjuvant IV liposomal doxorubicin to increase tumor destruction by 25–30% [130]. One possible mechanism for the improved treatment efficacy is due to increased vascular permeability as a result of endothelial thermal stress/injury. Kong et al. [126] demonstrated enlarged pores on vascular endothelial in tumors after mild hyperthermia. Other studies have also confirmed a correlation between large drug accumulations in tumor regions and high temperature rises in tumor models [131,132]. Li et al. [127] found large endothelial lining gaps (87 nm) after a whole-body heating at 41 °C for 30 min and deep penetration depths of large-sized liposomes from the vessel wall in the interstitial fluid space (up to 27.5 µm) [127]. Most importantly, this study did not demonstrate extravasation in normal tissues, so liposome delivery can specifically target tumors [127]. Another suggested mechanism is derived from a multi-drug resistance membrane protein that can be damaged by heat. Previous studies have demonstrated that reversible thermal damage to tumor vessels and cells would increase cellular or nuclear membrane permeability of large-molecule drugs, leading to more drug deposition [133,134,135,136,137,138].

Limited IFP reductions were reported in the tumors subject to local heating in previous experiments. On the contrary, large IFP decreases were shown in tumors after whole-body hyperthermia. Since the animals in these experiments were conscious during whole-body hyperthermia, these investigators suggested that thermoregulation may play an important role. It is well known that elevating body temperature would trigger thermoregulation. It was suggested that neurovascular agents may be released through central control and they travel through the bloodstream to reach the tumor site. These neurovascular agents may be a major reason influencing the IFP reductions [124,125]. Whole-body heating may also improve lymphatic drainage, thus increasing overall blood circulation [139,140]. Another possible effect of mild heating may be related to solid stress modification in tumors. Tumors typically exhibit high solid stress [103,141], leading to the compression of blood vessels and lymphatic vessels in tumors. If the solid stress could be decreased by mild heating, it would result in the opening of these vessels to improve fluid drainage and blood flow in tumors [142,143]. 

Theoretical simulations are useful tools to evaluate proposed transport mechanisms to explain experimental observations. Theoretical simulations of fluid transport and nanoparticle diffusion in tumors have been conducted since the 1980s. In these studies, fluid transport and diffusion in tumors were modeled as flow in a porous medium, while the transvascular flow was modeled as volumetric fluid sources and sinks. Nanoparticle diffusion and advection in the interstitial fluid space is modeled as a continuum convection and particle diffusion. An early study by Baxter and Jain investigated the effect of hydraulic conductivity on drug concentration in a 1D model in a spherical tumor [144]. Later theoretical simulations were expanded to be 2D or 3D with various tumor sizes and shapes [89]. These models were significantly improved recently by including cell uptakes of drugs or nanoparticles, and these approaches often required multiscale modeling simulation [145]. The accuracy of theoretical modeling was greatly improved as shown in the agreement between theoretical predictions and experimental measurements. In the study by Stepleton et al. [146], they reported predictions of liposome transport in tumors similar to that in computed tomography (CT) results. Some simulation predictions suggested that a small percentage decrease in IFP would significantly enhance nanoparticle delivery to tumors [122]. Another recent theoretical simulation investigated the extent to which increasing the lymphatic drainage or the permeability of a porous tumor would reduce the IFPs at the tumor central regions [139]. The authors reported a very good agreement between theoretical predictions and experimental measurements on the enhanced nanoparticle accumulation 24 h after injection. Although their results suggest the possible roles played by an increases in the hydraulic conductivity and/or recovery of lymphatic functions, experimental evidence is still needed with advanced engineering tools to measure these parameters [139]. 

## 5. Conclusions Remarks

Within the past decades, one has seen important advancements in the use of heating in many therapeutic procedures, especially for cancer treatment. Hyperthermia is often used either as a singular therapy leading to direct heat-induced cytotoxic response, and/or as an adjuvant therapy with radiation and drugs in cancer treatment. In recent years, due to advancement in nanotechnology in medicine, many experimental studies have demonstrated an interaction between heating and nanoparticles in tumors. Major experimental observations illustrated how heating changed the microenvironments in tumors via affecting transport properties related to nanoparticle depositions and distribution in tumors. High-intensity heating causes irreversible thermal damage to cancer tissue, and local damage may alter tumor porosity, thus leading to strong particle diffusion. Nanoparticle migration observed in experiments, on the other hand, requires including dynamic responses in theoretical modeling to design a treatment protocol with reliable predictive capability. In mild local or whole-body hyperthermia, heating may trigger thermoregulation in the entire body, increase blood perfusion in the tumor, alter transport properties, and possibly improve lymphatic drainage. The consequence is observed IFP reductions and enhanced nanoparticles delivered to tumors. The challenge facing engineers and clinicians in the field is that the observed effects of heating are limited to parameters at macro-scale levels including pressure, temperature, blood perfusion, etc. There is a lack of direct experimental evidence at the cellular or molecular levels. For example, nanoparticle migration is not directly measured during a heating session. Changes in tumor porosity, permeability, and lymphatic function have not been demonstrated in these experiments. Therefore, although some theoretical simulations showed good agreements with experimental measurements, these simulations were based on assumed transport mechanisms affected by local or whole-body hyperthermia. Future experimental studies are warranted to evaluate the underlying thermal and fluid dynamic mechanisms to provide direct experimental evidence of the influences. The current experiments on animal models may be adopted in future clinical studies. Mild whole-body heating within a short 1 h duration at 40 °C might be tolerated by patients and easily implemented and controlled [147,148,149]. High-intensity local heating in tumors could be used first to generate an easy passage for later systemic drug delivery. Multifunctional nanoparticles could be developed to serve both heating and drug carrying. Thermal damage to certain tumor regions would facilitate drugs to be delivered to cover the entire tumor. Manipulating the tumor microenvironment via local or whole-body hyperthermia has great potential in cancer treatment to enhance delivery of drug-carrying nanoparticles tumors or to achieve desirable nanoparticle distribution in tumor ablation. 

## References

[1] American Cancer Society (2024). Cancer Facts and Figures. https://www.cancer.org/research/cancer-facts-statistics/all-cancer-facts-figures/2024-cancer-facts-figures.html.

[2] Shuichi Miyamoto S., Shimono K. (2020). Molecular modeling to estimate the diffusioncoefficients of drugs and other small molecules. Molecules.

[3] Zhang A., Mi X., Yang G., Xu L.X. (2009). Numerical study of thermally targeted liposomal drug delivery in tumor. J. Heat Transf..

[4] Bala V.-M., Lampropoulou D.I., Grammatikaki S., Kouloulias V., Lagopati N., Aravantinos G., Gazouli M. (2024). Nanoparticle-mediated hyperthermia and cytotoxicity mechanisms in cancer. Int. J. Mol. Sci..

[5] Cherukuri P., Glazer E.S., Curley S.A. (2010). Targeted hyperthermia using metal nanoparticles. Adv. Drug Deliv. Rev..

[6] El-Sayed I., Huang X., Elsayed M. (2006). Selective laser photo-thermal therapy of epithelial carcinoma using anti-EGFR antibody conjugated gold nanoparticles. Cancer Lett..

[7] Hirsch L.R., Stafford R.J., Bankson J.A., Sershen S.R., Rivera B., Price R.E., Hazle J.D., Halas N.J., West J.L. (2003). Nanoshell-mediated near-infrared thermal therapy of tumors under magnetic resonance guidance. Proc. Natl. Acad. Sci. USA.

[8] Jain P.K., Lee K.S., El-Sayed I.H., and El-Sayed M.A. (2006). Calculated absorption and scattering properties of gold nanoparticles of different size, shape, and composition: applications in biological imaging and biomedicine. J. Phys. Chem. B.

[9] Jain S., Hirst D.G., O’Sullivan J.M. (2012). Gold nanoparticles as novel agents for cancer therapy. Br. J. Radiol..

[10] LeBrun A., Ma R., Zhu L. (2016). MicroCT Image based simulation to design heating protocols in magnetic nanoparticle hyperthermia for cancer treatment. J. Therm. Biol..

[11] LeBrun A., Joglekar T., Bieberich C., Ma R., Zhu L. (2017). Treatment efficacy for validating microCT-based theoretical simulation approach in magnetic nanoparticle hyperthermia for cancer treatment. J. Heat Transf..

[12] Shirvalilou S., Tavangari Z., Parsaei M.H., Sargazi S., Sheervalilou R., Shirvaliloo M., Ghaznavi H., Khoei S. (2023). The future opportunities and remaining challenges in the application of nanoparticle-mediated hyperthermia combined with chemo-radiotherapy in cancer. WIREs Nanomed. Nanotechnol..

[13] Cheheltani R., Ezzibdeh R.M., Chhour P., Pulaparthi K., Kim J., Jurcova M., Hsu J.C., Blundell C., Litt H.I., Ferrari V.A. (2016). Tunable, biodegradable gold nanoparticles as contrast agents for computed tomography and photoacoustic imaging. Biomaterials.

[14] Chen Q., Li K., Wen S., Liu H., Peng C., Cai H., Shen M., Zhang G., Shi X. (2013). Targeted CT/MR dual mode imaging of tumors using multifunctional dendrimer-entrapped gold nanoparticles. Biomaterials.

[15] Connor E.E., Mwamuka J., Gole A., Murphy C.J., Wyatt M.D. (2005). Gold nanoparticles are taken up by human cells but do not cause acute cytotoxicity. Small.

[16] Zhang J., Li C., Zhang X., Huo S., Jin S., An F.-F., Wang X., Xue X., Okeke C.I., Duan G. (2015). In vivo tumor-targeted dual-modal fluorescence/CT imaging using a nanoprobe co-loaded with an aggregation-induced emission dye and gold nanoparticles. Biomaterials.

[17] Yin B., Ho W.K.H.H., Xia X., Chan C.K.W.C., Zhang Q., Ng Y.M., Lam C.Y.K., Cheung J.C.W., Wang J., Yong M. (2023). A multilayered mesoporous gold nanoarchitecture for ultraeffective near-infrared light-controlled chemo/photothermal therapy for cancer guided by SERS imaging. Small.

[18] Gobin A.M., Moon J.J., West J.L. (2008). EphrinA I-targeted nanoshells for photothermal ablation of prostate cancer cells. Int. J. Nanomed..

[19] Huang X., Jiang P., Tanaka T. (2011). A review of dielectric polymer composites with high thermal conductivity. IEEE Electr. Insul. Mag..

[20] Loo C., Lowery A., Halas N., West J., Drezek R. (2005). Immunotargeted nanoshells for integrated cancer imaging and therapy. Nano Lett..

[21] Yang H.-W., Liu H.-L., Li M.-L., His I.W., Fan C.-T., Huang C.-Y., Lu Y.-J., Hua M.-Y., Chou H.-Y., Liaw J.-W. (2013). Magnetic gold-nanorod/PNIPAAmMA nanoparticles for dual magnetic resonance and photoacoustic imaging and targeted photothermal therapy. Biomaterials.

[22] Espinosa A., Di Corato R., Kolosnjaj-Tabi J., Flaud P., Pellegrino T., Wilhelm C. (2016). Duality of iron oxide nanoparticles in cancer therapy: Amplification of heating efficiency by magnetic hyperthermia and photothermal bimodal treatment. ACS Nano.

[23] Hoopes J., Mazur M., Osterberg B., Song A., Gladstone J., Steinmetz F., Fiering N. (2017). Effect of intra-tumoral magnetic nanoparticle hyperthermia and viral nanoparticle immunogenicity on primary and metastatic cancer. Energy-Based Treat. Tissue Assess. IX.

[24] LeBrun A., Zhu L., Shrivastava D. (2018). Magnetic Nanoparticle Hyperthermia in Cancer Treatment: History, Mechanism, Imaging-Assisted Protocol Design, and Challenges. Theory and Applications of Heat Transfer in Humans.

[25] Maier-Hauff K., Ulrich F., Nestler D., Niehoff H., Wust P., Thiesen B., Orawa H., Budach V., Jordan A. (2010). Efficacy and safety of intratumoral thermotherapy using magnetic iron-oxide nanoparticles combined with external beam radiotherapy on patients with recurrent glioblastoma multiforme. J. Neuro-Oncol..

[26] Petryk A., Misra A., Mazur C.M., Petryk J.D., Hoopes P.J. (2015). Magnetic nanoparticle hyperthermia cancer treatment efficacy dependence on cellular and tissue level particle concentration and particle heating properties. Energy-Based Treatment of Tissue and Assessment VIII.

[27] Davarakonda S.B., Myers M.R., Lanier M., Dumoulin C., Banerjee R.K. (2017). Assessment of gold nanoparticle-mediated-enhanced hyperthermia using MR-guided high-intensity focused ultrasound ablation procedure. Nano Lett..

[28] Devarakonda S.B., Stringer K., Rao M., Myers M., Banerjee R. (2019). Assessment of enhanced thermal effect due to gold nanoparticles during mr-guided high-intensity focused ultrasound (hifu) procedures using a mouse-tumor model. ACS Biomater. Sci. Eng..

[29] Knanal N., Marciniak M., Daniel M.-C., Zhu L., Lanier M., Dumoulin C., Banerjee R.K. Functionalized nanoparticles mediated high intensity focused ultrasound (HIFU) ablation in mice. Proceedings of the Summer Biomechanics, Bioengineering and Biotransport Conference.

[30] Hosseinpour A., Soltani M., Souri M. (2024). Improving tumor treatment through intratumoral injection of drug-loaded magnetic nanoparticles and low-intensity ultrasound. Sci. Rep..

[31] Souri M., Moradi Kashkooli F., Soltani M. (2022). Analysis of magneto-hyperthermia duration in nano-sized drug delivery system to solid tumors using intravascular-triggered thermosensitive-liposome. Pharm. Res..

[32] Zhan W., Gedroyc W., Xu X.Y. (2019). Towards a multiphysics modelling framework for thermosensitive liposomal drug delivery to solid tumour combined with focused ultrasound hyperthermia. Biophys. Rep..

[33] Dockery L., Zalesak-Kravec S., Kane M.A., Daniel M.-C. (2022). Modular and efficient synthesis of a poly (propylene imine) (PPI) dendron applied to acid-sensitive doxorubicin conjugation. Tetrahedron.

[34] Dockery L., Daniel M.C. (2018). Dendronized systems for the delivery of chemotherapeutics. Adv. Cancer Res..

[35] Dreher M.R., Liu W., Michelich C.R., Dewhirst M.W., Yuan F., Chilkoti A. (2006). Tumor vascular permeability, accumulation, and penetration of macromolecular drug carriers. Int. J. Rad. Onc. Biol. Phys..

[36] Salavati H., Debbaut C., Pullens P., Ceelen W. (2022). Interstitial fluid pressure as an emerging biomarker in solid tumors. Biochim. Biophys. Acta (BBA) Rev. Cancer.

[37] Hergt R., Dutz S., Müller R., Zeisberger M. (2006). Magnetic particle hyperthermia: Nanoparticle magnetism and materials development for cancer therapy. J. Phys. Condens. Matter.

[38] Link S., El-Sayed M.A. (2000). Shape and size dependence of radiative, non-radiative and photothermal properties of gold nanocrystals. Int. Rev. Phys. Chem..

[39] Link S., Mohamed M.B., El-Sayed M.A. (1999). Simulation of the optical absorption spectra of gold nanorods as a function of their aspect ratio and the effect of the medium dielectric constant. J. Phys. Chem. B.

[40] Maeda H. (2001). The enhanced permeability and retention (EPR) effect in tumor vasculature: The key role of tumor-selective macromolecular drug targeting. Adv. Enzym. Regul..

[41] Sofou S. (2008). Radionuclide carriers for targeting of cancer. Int. J. Nanomed..

[42] Xiang Y., Chen Q., Nan Y., Liu M., Xiao Z., Yang Y., Zhang J., Ying X., Long X., Wang S. (2024). Nitric Oxide-based nanomedicines for conquering TME fortress: Say ”no” to insufficient tumor treatment. Adv. Funct. Mater..

[43] Siafaka P.I., Okur N.U., Karavas E., Bikiaris D.N. (2016). Surface modified multifunctional and stimuli responsive nanoparticles for drug targeting: Current status and uses. Int. J. Mol. Sci..

[44] Kato Y., Ozawa S., Miyamoto C., Maehata Y., Suzuki A., Maeda T., Baba Y. (2013). Acidic extracellular microenvironment and cancer. Cancer Cell Int..

[45] Kraft J.C., Freeling J.P., Wang Z., Ho R.J. (2014). Emerging Research and clinical development trends of liposome and lipid nanoparticle drug delivery systems. J. Pharm. Sci..

[46] Farokhzad O.C., Cheng J., Teply B.A., Sherifi I., Jon S., Kantoff P.W., Richie J.P., Langer R. (2006). Targeted nanoparticle-aptamer bioconjugates for cancer chemotherapy in vivo. Proc. Natl. Acad. Sci. USA.

[47] Tong R., Cheng J. (2007). Anticancer polymeric nanomedicines. Polym. Rev..

[48] Torchilin V.P. (2006). Micellar Nanocarriers: Pharmaceutical perspectives. Pharm. Res..

[49] Jannin V., Musakhanian J., Marchaud D. (2008). Approaches for the development of solid and semi-solid lipid-based formulations. Adv. Drug Deliv. Rev..

[50] Giocondi M., Pacheco L., Milhiet P.E., Le Grimellec C. (2001). Temperature dependence of the topology of supported dimirystoyl–distearoyl phosphatidylcholine bilayers. Ultramicroscopy.

[51] Tran S., DeGiovanni P., Piel B., Rai P. (2017). Cancer nanomedicine: A review of recent success in drug delivery. Clin. Transl. Med..

[52] Colombo P., Bettini R., Santi P., Peppas N.A. (2000). Swellable matrices for controlled drug delivery: Gel-layer behaviour, mechanisms and optimal performance. Pharm. Sci. Technol. Today.

[53] Joseph M., Trinh H.M., Cholkar K., Pal D., Mitra A.K. (2016). Recent perspectives on the delivery of biologics to back of the eye. Expert Opin. Drug Deliv..

[54] Nune S.K., Gunda P., Thallapally P.K., Lin Y., Laird M., Berkland C.J. (2009). Nanoparticles for biomedical imaging. Expert Opin. Drug Deliv..

[55] Behrouzkia Z., Joveini Z., Keshavarzi B., Eyvazzadeh N., Aghdam R.Z. (2016). Hyperthermia: How can it be used?. Oman Med. J..

[56] Ye H., De S. (2017). Thermal injury of skin and subcutaneous tissues: A review of experimental approaches and numerical models. Burn. J. Int. Soc. Burn Inj..

[57] Giustini A.J., Petryk A.A., Cassim S.M., Tate J.A., Baker I., Hoopes P.J. (2010). Magnetic nanoparticle hyperthermia in cancer treatment. Nano Life.

[58] Kolhatkar A., Jamison A., Litvinov D., Willson R., Lee T. (2013). Tuning the Magnetic Properties of Nanoparticles. Int. J. Mol. Sci..

[59] LeBrun A., Joglekar T., Bieberich C., Ma R., Zhu L. (2016). Identification of infusion strategy for achieving repeatable nanoparticle distribution and quantification of thermal dosage using micro-CT Hounsfield unit in magnetic nanoparticle hyperthermia. Int. J. Hyperth..

[60] Rytov R.A., Bautin V.A., Usov N.A. (2022). Towards optimal thermal distribution in magnetic hyperthermia. Sci. Rep..

[61] Guibert C., Dupuis V., Peyre V., Fresnais J. (2015). Hyperthermia of magnetic nanoparticles: Experimental study of the role of aggregation. J. Phys. Chem. C.

[62] Pennes H.H. (1948). Analysis of tissue and arterial blood temperatures in the resting human forearm. J. Appl. Physiol..

[63] Jordan A., Wust P., Fählin H., John W., Hinz A., Felix R. (1993). Inductive heating of ferrimagnetic particles and magnetic fluids: Physical evaluation of their potential for hyperthermia. Int. J. Hyperth..

[64] Etheridge M.L., Hurley K.R., Zhang J., Jeon S., Ring H.L., Hogan C., Haynes C., Garwood M., Bischof J.C. (2014). Accounting for biological aggregation in heating and imaging of magnetic nanoparticles. Technology.

[65] Etheridge M., Manuchehrabadi N., Franklin R., Bischof J. (2016). Superparamagnetic Iron Oxide Nanoparticle Heating. Nanoparticle Heat Transfer and Fluid Flow.

[66] Rosensweig R. (2002). Heating magnetic fluid with alternating magnetic field. J. Magn. Magn. Mater..

[67] Wust P., Nadobny J., Fähling H., Jordan A., Felix R., Breit A., Heuck A., Lukas P., Kneschaurek P., Mayr M. (1992). Code Comparison and verification for patient-specific three-dimensional treatment planning in regional hyperthermia. Tumor Response Monitoring and Treatment Planning.

[68] Wust P., Gneveckow U., Johannsen M., Böhmer D., Henkel T., Kahmann F., Sehouli J., Felix R., Ricke J., Jordan A. (2006). Magnetic nanoparticles for interstitial thermotherapy—Feasibility, tolerance and achieved temperatures. Int. J. Hyperth..

[69] Kalambur V.S., Longmire E.K., Bischof J.C. (2007). Cellular level loading and heating of superparamagnetic iron oxide nanoparticles. Langmuir.

[70] Beola L., Grazu V., Fernandez-Afonso Y., Fratila R.M., de las Heras M., de la Fuente J.M., Gutierrez L., Asin L. (2021). Critical parameters to improve pancreatic cancer treatment using magnetic hyperthermia: Field conditions, immune response, and particle biodistribution. ACS Appl. Mater. Interfaces.

[71] Vassallo M., Martella D., Barrera G., Celegato F., Coisson M., Ferrero R., Olivetti E.S., Troia A., Sozero H., Parmaggiani C. (2023). Improvement of hyperthermia properties of iron oxide nanoparticles by surface coating. ACS Omega.

[72] Sheervalilou R., Khoei S., Khoee S., Shirvaliloo M., Sadri E., Shirvalilou S., Goudarzi M. (2023). Magnetohyperthermia-synergistic glioma cancer therapy enabled by magnetic graphene oxide nanoheaters: Promising nanostructure for in vitro and in vivo applications. Cancer Nano.

[73] Lee J.H., Kim B., Kim Y., Kim S.K. (2021). Ultra-high rate of temperature increment from superparamagnetic nanoparticles for highly efficient hyperthermia. Sci. Rep..

[74] Zhao Q., Wang L., Cheng R., Mao L., Arnold R.D., Howerth E.W., Chen Z., Platt S. (2012). Magnetic nanoparticle-based hyperthermia for head and neck cancer in mouse models. Theranostics.

[75] He M., Cao X.C., He G.C., Sheng X.F., Ai X.H., Wu Y.H. (2014). Casticin inhibits epithelial-mesenchymal transition of liver cancer stem cells of the SMMC-7721 cell line through downregulating Twist. Oncol. Lett..

[76] Attaluri A., Ma R., Qiu Y., Li W., Zhu L. (2011). Nanoparticle distribution and temperature elevations in prostate tumors in mice during magnetic nanoparticle hyperthermia. Int. J. Hyperth..

[77] Johannsen M., Gneveckow U., Eckelt L., Feussner A., WaldÖFner N., Scholz R., Deger S., Wust P., Loening S., Jordan A. (2005). Clinical hyperthermia of prostate cancer using magnetic nanoparticles: Presentation of a new interstitial technique. Int. J. Hyperth..

[78] Attaluri A., Kandala S.K., Zhou H., Wabler M., DeWeese T.L., Ivkov R. (2020). Magnetic nanoparticle hyperthermia for treating locally advanced unresectable and borderline resectable pancreatic cancers: The role of tumor size and eddy-current heating. Int. J. Hyperth..

[79] Li W., Liu Y., Qian Z., Yang Y. (2017). Evaluation of tumor treatment of magnetic nanoparticles driven by extremely low frequency magnetic field. Sci. Rep..

[80] Gu Q., Min Zaw M., Munuhe T., Ma R., Zhu L. Nanoparticle re-distribution in tissue-equivalent gels induced by magnetic nanoparticle hyperthermia. Proceedings of the Summer Biomechanics, Bioengineering, & Biotransport Conference.

[81] Gu Q. (2019). Heating Induced Nanoparticle Redistribution in PC3 Tumors: In Vivo Experiments and MicroCT Imaging Analyses. Ph.D. Thesis.

[82] Gu Q., Joglekar T., Bieberich C., Ma R., Zhu L. (2019). Nanoparticle redistribution in PC3 tumors induced by local heating in magnetic nanoparticle hyperthermia: In vivo experimental study. J. Heat Transf..

[83] Wankhede M., Bouras A., Kaluzova M., Hadjipanayis C. (2012). Magnetic nanoparticles: An emerging technology for malignant brain tumor imaging and therapy. Expert Rev. Clin. Pharmacol..

[84] Gunakala S.R., Job V.M., Murthy P.V.S.N., Sibanda P., Raju C.S.K. (2024). Mathematical model of magnetic hyperthermia therapy for breast tumour via intratumoural injection of iron–platinum nanoparticles. Case Stud. Therm. Eng..

[85] Xu C., Miranda-Nieves D., Ankrum J.A., Matthiesen M.E., Phillips J.A., Roes I., Wojtkiewicz G., Juneja V., Kultima J., Zhao W. (2012). Tracking mesenchymal stem cells with iron oxide nanoparticle loaded poly(lactide-co-glycolide) microparticles. Nano Lett..

[86] Comerford S., Huang Z., Du X., Wang Y., Cai L., Witkiewicz A., Walters H., Tantawy M., Fu A., Manning H. (2014). Acetate dependence of tumors. Cell.

[87] Costello J.T., Culligan K., Selfe J., Donnelly A.E. (2012). Muscle, skin and core temperature after −110 °C cold air and 8 °C water treatment. PLoS ONE.

[88] Singh M., Ma R., Zhu L. (2021). Quantitative evaluation of effects of coupled temperature elevation, thermal damage, and enlarged porosity on nanoparticle migration in tumors during magnetic nanoparticle hyperthermia. Int. Commun. Heat Mass Transf..

[89] El-Kareh A.W., Secomb T.W. (1995). Effect of increasing vascular hydraulic conductivity on delivery of macromolecular drugs to tumor cells. Int. J. Radiat. Oncol. Biol. Phys..

[90] Mahesh N., Singh N., Talukdar P. (2023). A mathematical model of intratumoral infusion, particle distribution and heat transfer in cancer tumors: In-silico investigation of magnetic nanoparticle hyperthermia. Int. J. Therm. Sci..

[91] Truskey G.A., Yuan F., Katz D.F. (2009). Transport Phenomena in Biological Systems.

[92] De J. (2008). Drug delivery and nanoparticles: Applications and hazards. Int. J. Nanomed..

[93] Johnson E.R., Matthay M.A. (2010). Acute lung injury: Epidemiology, pathogenesis, and treatment. J. Aerosol Med. Pulm. Drug Deliv..

[94] Singh R., Lillard J.W. (2009). Nanoparticle-based targeted drug delivery. Exp. Mol. Pathol..

[95] Izci M., Maksoudian C., Manshian B.B., Soenen S.J. (2021). The use of alternative strategies for enhanced nanoparticle delivery to solid tumors. ACS Chem. Rev..

[96] Oh H., Jun D.W., Saeed W.K., Nguyen M.H. (2016). Non-alcoholic fatty liver diseases: Update on the challenge of diagnosis and treatment. Clin. Mol. Hepatol..

[97] Li S., Li L., Lin X., Chen C., Luo C., Huang Y. (2022). Targeted inhibition of tumor inflammation and tumor-platelet crosstalk by nanoparticle-mediated drug delivery mitigates cancer metastasis. ACS Nano.

[98] Steichen S.D., Caldorera-Moore M., Peppas N.A. (2013). A review of current nanoparticle and targeting moieties for the delivery of cancer therapeutics. Eur. J. Pharm. Sci. Off. J. Eur. Fed. Pharm. Sci..

[99] Scott M.C., Chen C.C., Mecklenburg M., Zhu C., Xu R., Ercius P., Dahmen U., Regan B.C., Miao J. (2012). Electron tomography at 2.4-Angstrom resolution. Nature.

[100] Jackson D., Gooya J., Mao S., Kinneer K., Xu L., Camara M., Fazenbaker C., Fleming R., Swamynathan S., Meyer D. (2008). A human antibody-drug conjugate targeting EphA2 inhibits tumor growth in vivo. Cancer Res..

[101] Rodzinski A., Guduru R., Liang P., Hadjikhani A., Stewart T., Stimphil E., Runowica C., Cote E., Altman N., Datar R. (2016). Targeted and controlled anticancer drug delivery and release with magnetoelectric nanoparticles. Sci. Rep..

[102] Nagy J.A., Chang S., Dvorak A.M., Dvorak H.F. (2009). Why are tumour blood vessels abnormal and why is it important to know?. Br. J. Cancer.

[103] Stylianopoulos T., Martin J.D., Chauhan V.P., Jain R.K., Diop-Frimpong B., Bardeesy N., Smith B.L., Ferrone C.R., Hornicek F.J., Boucher Y. (2012). Causes, consequences, and remedies for growth-induced solid stress in murine and human tumors. Proc. Natl. Acad. Sci. USA.

[104] Bockhorn M., Jain R.K., Munn L.L. (2007). Active versus passive mechanisms in metastasis: Do cancer cells crawl into vessels, or are they pushed?. Lancet Oncol..

[105] Pardridge W.M. (2012). Drug transport across the blood–brain barrier. J. Cereb. Blood Flow Metab..

[106] Hladky S.B., Barrand M.A. (2014). Mechanisms of fluid movement into, through and out of the brain: Evaluation of the evidence. Fluids Barriers CNS.

[107] Margaris K.N., Black R.A. (2012). Modelling the lymphatic system: Challenges and opportunities. J. R. Soc. Interface.

[108] Nacev A., Beni C., Bruno O., Shapiro B. (2010). Magnetic nanoparticle transport within flowing blood and into surrounding tissue. Nanomedicine.

[109] Paliwal R., Babu R.J., Palakurthi S. (2014). Nanomedicine scale-up technologies: Feasibilities and challenges. AAPS Pharm. Sci. Technol..

[110] Woo J.R., Liss M.A., Muldong M.T., Palazzi K., Strasner A., Ammirante M., Varki N., Shabaik A., Howell S., Kane C. (2014). Tumor infiltrating B-cells are increased in prostate cancer tissue. J. Transl. Med..

[111] Baum J., Duffy H.S. (2011). Fibroblasts and myofibroblasts: What are we talking about?. J. Cardiovasc. Pharmacol..

[112] Jain R.K., Martin J.D., Stylianopoulos T. (2014). The role of mechanical forces in tumor growth and therapy. Annu. Rev. Biomed. Eng..

[113] Stylianopoulos T., Munn L.L., Jain R.K. (2018). Reengineering the physical microenvironment of tumors to improve drug delivery and efficacy: From mathematical modeling to bench to bedside. Trends Cancer.

[114] Wilhelm S., Tavares A.J., Dai Q., Ohta S., Audet J., Dvorak H.F., Chan W.C.W. (2016). Analysis of nanoparticle delivery to tumours. Nat. Rev. Mater..

[115] Song C.W. (1984). Effect of local hyperthermia on blood flow and microenvironment: A review. Cancer Res..

[116] Hauck M.L., Dewhirst M.W., Bigner D.D., Zalutsky M.R. (1997). Local hyperthermia improves uptake of a chimeric monoclonal antibody in a subcutaneous xenograft model. Clin. Cancer Res..

[117] Lammers T., Peschke P., Kühnlein R., Subr V., Ulbrich K., Debus J., Huber P., Hennink W., Storm G. (2007). Effect of radiotherapy and hyperthermia on the tumor accumulation of HPMA copolymer-based drug delivery system. J. Control. Release.

[118] Gu Q., Liu S., Ray A.S., Florinas S., Christie R.J., Daniel M.-C., Bieberich C., Ma R., Zhu L. (2020). Mild whole body hyperthermia induced interstitial fluid pressure (IFP) reduction and enhanced nanoparticle delivery to PC3 tumours: In vivo studies and microCT analyses. ASME J. Therm. Sci. Eng. Appl..

[119] Fan F., Xie B., Yang L. (2021). Promoting nanoparticle delivery efficiency to tumors by locally increasing blood flow there. ACS Appl. Bio Mater..

[120] Zhang H., Xu J., Gao B., Wang H., Huang J., Zhou J., Yang R., Yan F., Peng Y. (2021). Synergistic cascade strategy based on modifying tumor microenvironment for enhanced breast cancer therapy. Front. Pharmacol..

[121] Leunig M., Goetz A.E., Dellian M., Zetterer G., Gamarra F., Jain R.K., Messmer K. (1992). Interstitial fluid pressure in solid tumors following hyperthermia: Possible correlation with therapeutic response. Cancer Res..

[122] Stepleton S., Dunne M., Milosevic M., Tran C.W., Gold M.J., Vedadi A., Mckee T.D., Ohashi P.S., Allen C., Jaffray D.A. (2018). Radiation and heat improve the delivery and efficacy of nanotherapeutics by modulating intratumoral fluid dynamic. ACS Nano.

[123] Hauck M.L., Coffin D.O., Dodge R.K., Dewhirst M.W., Mitchell J.B., Zalutsky M.R. (1997). A local hyperthermia treatment which enhances antibody uptake in a glioma xenograft model does not affect tumor interstitial fluid pressure. Int. J. Hyperth..

[124] Sen A., Capitano M., Spernyak J.A., Schueckler J., Thomas S., Singh A., Evans S.S., Hylander B.L., Repasky E.A. (2011). Mild elevation of body temperature reduces tumor interstitial fluid pressure and hypoxia, and enhances efficacy of radiotherapy in murine tumor models. Cancer Res..

[125] Winslow T.B., Eranki A., Ullas S., Singh A.K., Repasky E.A., Sen A. (2015). A pilot study of the effects of mild systemic heating on human head and neck tumour xenografts: Analysis of tumour perfusion, interstitial fluid pressure, hypoxia and efficacy of radiation therapy. Int. J. Hyperth..

[126] Koning G.A., Eggermont A.M.M., Lindner L.H., ten Hagen T.L.M. (2010). Hyperthermia and thermosensitive liposomes for improved delivery of chemotherapeutic drugs to solid tumors. Pharm. Res..

[127] Li L., ten Hagen T.L.M., Bolkestein M., Gasselhuber A., Yatvin J., van Rhoon G.C., Eggermont A.M.M., Haemmerich D., Koning G.A. (2013). Improved intratumoral nanoparticle extravasation and penetration by mild hyperthermia. J. Control. Release.

[128] Schiffelers R.M., Koning G.A., ten Hagen T.L.M., Fens M.H.A.M., Schraa A.J., Janssen A.P.C.A., Kok R.J., Molema G., Storm G. (2003). Anti-tumor efficacy of tumor vasculature-targeted liposomal doxorubicin. J. Control. Release.

[129] Yuan F., Dellian M., Fukumura D., Leunig M., Berk D.A., Torchilin V., Jain R.K. (1995). Vascular permeability in a human tumor xenograft: Molecular size dependence and cutoff size. Cancer Res..

[130] Ahmed M., Goldberg S.N. (2004). Combination radiofrequency thermal ablation and adjuvant IV liposomal doxorubicin increases tissue coagulation and intratumoural drug accumulation. Int. J. Hyperth..

[131] Ahmed M., Liu Z., Horkan C., Tochilin V.P., Lukyanov A.N., Goldberg S.N. (2003). Raido-frequency tumor ablation combined with intravenous liposomal doxorubicin increases tumor coagulation and intratumoral drug accumulation in a large animal tumor model. Radiology.

[132] Monsky W.L., Kruskal J.B., Lukyanov A.N., Girnun G.D., Ahmed M., Gazelle G.S., Huertas J.C., Stuart K.E., Torchilin V.P., Goldberg S.N. (2002). Radio-frequency ablation increases intratumoral liposomal doxorubicin accumulation in a rat breast tumor model. Radiology.

[133] Kawai H., Minamiya Y., Kitamura M., Matsuzaki I., Hashimoto M., Suzuki H., Abo S. (1997). Direct measurement of doxorubicin concentration in the intact, living single cancer cell during hyperthermia. Cancer.

[134] Merlin J.L., Marchal S., Ramacci C., Notter D., Vigneron C. (1993). Antiproliferative activity of thermosensitive liposome-encapsulated doxorubicin combined with 43 °C hyperthermia in sensitive and multidrug-resistant MCF-7 cells. Eur. J. Cancer.

[135] Osborne E.J., MacKillop W.J. (1987). The effect of exposure to elevated temperatures on membrane permeability to adriamycin in Chinese hamster ovary cells in vitro. Cancer Lett..

[136] Dunne M., Regenold M., Allen C. (2020). Hyperthermia can alter tumor physiology and improve chemo- and radio-therapy efficacy. Adv. Drug Deliv. Rev..

[137] Song C.W., Park H.J., Lee C.K., Griffin R. (2005). Implications of increased tumor blood flow and oxygenation caused by mild temperature hyperthermia in tumor treatment. Int. J. Hyperth..

[138] Toffoli G., Bevilacqua C., Franceschin A., Boiocchi M. (1989). Effect of hyperthermia on intracellular drug accumulation and chemosensitivity in drug-sensitive and drug-resistant P388 leukaemia cell lines. Int. J. Hyperth..

[139] Manpreet S., Ma R., Zhu L. (2021). Theoretical evaluation of enhanced gold nanoparticle delivery to PC3 tumors due to increased hydraulic conductivity or recovered lymphatic function after mild whole body hyperthermia. Med. Biol. Eng. Comput..

[140] Mariana V.F., Maria de Fátima G.G., Maria P. (2011). The effect of mechanical lymph drainage accompanied with heat on lymphedema. J. Res. Med. Sci..

[141] Allabashi R., Stach W., de la Escosura-Muñiz A., Liste Calleja L., Merkoçi A. (2008). ICP-MS: A powerful technique for quantitative determination of gold nanoparticles without previous dissolving. J. Nanoparticle Res..

[142] Leu A.J., Berk D.A., Lymboussaki A., Alitalo K., Jain R.K. (2000). Absence of functional lymphatics within a murine sarcoma: A molecular and functional evaluation. Cancer Res..

[143] Baxter L.T., Jain R.K. (1988). Vascular permeability and interstitial diffusion in superfused tissue: A two-dimensional model. Microvasc. Res..

[144] Baxter L.T., Jain R.K. (1989). Transport of fluid and macromolecules in tumors I. role of interstitial pressure and convection. Microvasc. Res..

[145] Su D., Ma R., Salloum M., Zhu L. (2010). Multi-scale study of nanoparticle transport and deposition in tissues during an injection process. Med. Biol. Eng. Comput..

[146] Stapleton S., Milosevic M., Allen C., Zheng J., Dunne M., Yeung I., Jaffray D.A. (2013). A mathematical model of the enhanced permeability and retention effect for liposome transport in solid tumors. PLoS ONE.

[147] Kockelmann F., Giger-Pabst U., Ouaissi M., Bucur P., Barbey S., von Ardenne A., Zieren J. (2024). First clinical safety and feasibility data of whole-body hyperthermia pressurized intraperitoneal aerosol chemotherapy (wbh-pipac) for peritoneal surface malignancies. Anticancer Res..

[148] Chia D.K.A., Demuytere J., Ernst S., Salavati H., Ceelen W. (2023). Effects of hyperthermia and hyperthermic intraperitoneal chemoperfusion on the peritoneal and tumor immune contexture. Cancers.

[149] Moghaddam F.F., Bakhshandeh M., Mofid B., Sahinbas H., Faeghi F., Mirzaei H., Rakhsha A., Kashi A.S.Y., Sadeghi R., Mahdavi A. (2024). Clinical effectiveness of combined whole body hyperthermia and external beam radiation therapy (EBRT) versus EBRT alone in patients with painful bony metastases: A phase III clinical trial study. J. Therm. Biol..

